# A Derivative of the D5 Monoclonal Antibody That Targets the gp41 N-Heptad Repeat of HIV-1 with Broad Tier-2-Neutralizing Activity

**DOI:** 10.1128/JVI.02350-20

**Published:** 2021-07-12

**Authors:** Adonis A. Rubio, Maria V. Filsinger Interrante, Benjamin N. Bell, Clayton L. Brown, Theodora U. J. Bruun, Celia C. LaBranche, David C. Montefiori, Peter S. Kim

**Affiliations:** a Stanford ChEM-H, Stanford University, Stanford, California, USA; b Department of Biology, Stanford University School of Humanities & Sciences, Stanford, California, USA; c Stanford Biophysics Program, Stanford University School of Medicine, Stanford, California, USA; d Stanford Medical Scientist Training Program, Stanford University School of Medicine, Stanford, California, USA; e Department of Molecular and Cellular Physiology, Stanford University School of Medicine, Stanford, California, USA; f Department of Biochemistry, Stanford University School of Medicine, Stanford, California, USA; g Department of Surgery, Duke University Medical Center, Durham, North Carolina, USA; h Duke Human Vaccine Institute, Duke University Medical Center, Durham, North Carolina, USA; i Chan Zuckerberg Biohub, San Francisco, California, USA; Ulm University Medical Center

**Keywords:** HIV-1, antibodies, prehairpin intermediate, virus

## Abstract

HIV-1 infection is initiated by the viral glycoprotein Env, which, after interaction with cellular coreceptors, adopts a transient conformation known as the prehairpin intermediate (PHI). The N-heptad repeat (NHR) is a highly conserved region of gp41 exposed in the PHI; it is the target of the FDA-approved drug enfuvirtide and of neutralizing monoclonal antibodies (mAbs). However, to date, these mAbs have only been weakly effective against tier-1 HIV-1 strains, which are most sensitive to neutralizing antibodies. Here, we engineered and tested 11 IgG variants of D5, an anti-NHR mAb, by recombining previously described mutations in four of D5’s six antibody complementarity-determining regions. One variant, D5_AR, demonstrated 6-fold enhancement in the 50% inhibitory dose (ID_50_) against lentivirus pseudotyped with HXB2 Env. D5_AR exhibited weak cross-clade neutralizing activity against a diverse set of tier-2 HIV-1 viruses, which are less sensitive to neutralizing antibodies than tier-1 viruses and are the target of current antibody-based vaccine efforts. In addition, the neutralization potency of D5_AR IgG was greatly enhanced in target cells expressing FcγRI, with ID_50_ values of <0.1 μg/ml; this immunoglobulin receptor is expressed on macrophages and dendritic cells, which are implicated in the early stages of HIV-1 infection of mucosal surfaces. D5 and D5_AR have equivalent neutralization potency in IgG, Fab, and single-chain variable-fragment (scFv) formats, indicating that neutralization is not impacted by steric hindrance. Taken together, these results provide support for vaccine strategies that target the PHI by eliciting antibodies against the gp41 NHR and support investigation of anti-NHR mAbs in nonhuman primate passive immunization studies.

**IMPORTANCE** Despite advances in antiretroviral therapy, HIV remains a global epidemic and has claimed more than 32 million lives. Accordingly, developing an effective HIV vaccine remains an urgent public health need. The gp41 N-heptad repeat (NHR) of the HIV-1 prehairpin intermediate (PHI) is highly conserved (>90%) and is inhibited by the FDA-approved drug enfuvirtide, making it an attractive vaccine target. However, to date, anti-NHR antibodies have not been potent. Here, we engineered D5_AR, a more potent variant of the anti-NHR antibody D5, and established its ability to inhibit HIV-1 strains that are more difficult to neutralize and are more representative of circulating strains (tier-2 strains). The neutralizing activity of D5_AR was greatly potentiated in cells expressing FcγRI; FcγRI is expressed on cells that are implicated at the earliest stages of sexual HIV-1 transmission. Taken together, these results bolster efforts to target the gp41 NHR and the PHI for vaccine development.

## INTRODUCTION

More than 35 million people currently live with HIV/AIDS globally (https://www.who.int/news-room/fact-sheets/detail/hiv-aids); despite the promise of antiretroviral therapy, a preventative HIV-1 vaccine remains an urgent global health need. However, after more than three decades of intense research, a prophylactic HIV/AIDS vaccine remains elusive. The RV144 trial raised expectations in 2009 (1), but the lack of efficacy reported in 2020 for the HVTN702 trial in South Africa, which was based on the prime-boost regimen utilized in RV144, was another major setback for the field (2).

Many current HIV-1 vaccine-directed research efforts are motivated by the finding that rare broadly neutralizing antibodies (bNAbs) can be isolated from patients chronically infected with HIV-1 (3–22). The main epitopes of these bNAbs include the CD4 binding site (CD4bs), the membrane proximal external region (MPER), the V1/V2 region, and the V3 glycan (23–26). Importantly, bNAbs are generally elicited as a result of complex coevolutionary pathways with viral variants and thus tend to be characterized by extensive somatic hypermutation (SHM) of the antibody genes. Indeed, germline precursors of bNAbs typically do not bind to most Env proteins (7, 27, 28). As a result, germline-lineage approaches (5, 10, 12, 15, 16, 21, 22) have emerged wherein an engineered immunogen is used to stimulate a germline precursor of a bNAb, followed by multistage multicomponent immunization aimed to direct affinity maturation toward the desired bNAb. While some progress has been made in eliciting bNAb-like responses in nonhuman primates (NHPs) (29–31), the technical and biological difficulties in eliciting rare bNAbs represent critical barriers to developing an antibody-based HIV vaccine against the native prefusion conformation of Env.

In contrast to these bNAb epitopes on the native conformation of Env, one highly conserved region of HIV-1 gp41 is exposed in a transient form of Env that is critical for viral infection, the prehairpin intermediate (PHI). Upon binding cellular receptors, Env undergoes substantial conformational changes to form the PHI, in which the N-heptad repeat (NHR) and C-heptad repeat (CHR) regions are exposed before the cellular and viral membranes come together for membrane fusion (32–34). Due to its high sequence conservation (∼93%) (https://www.hiv.lanl.gov/content/sequence/HIV/mainpage.html) and critical role in viral entry (32), the NHR is a promising target for blocking HIV-1 infection. Several inhibitors of HIV-1 infection that target the NHR and prevent viral membrane fusion have been identified (35–48). Cyclic d-peptide inhibitors that target the NHR have been used to prevent and treat simian-human immunodeficiency virus (SHIV) infection in rhesus macaques (35, 49). Most notably, the FDA-approved HIV-1 fusion inhibitor enfuvirtide (50–52) validates the NHR as a therapeutic target in humans.

**FIG 1:**
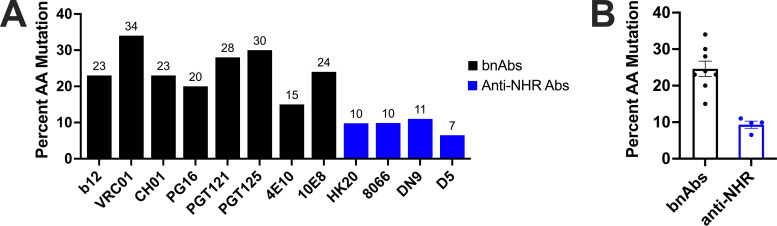
Anti-NHR antibodies have a lower level of somatic hypermutation than HIV-1 bNAbs. (A) Percentages of mutated amino acids across both heavy- and light-chain V genes from germline are reported for eight bNAbs (black) representative of the four main bNAb epitopes (MPER, V1/V2 region, V3 glycan, and CD4bs) and four monoclonal antibodies against the NHR (blue). Sequences of the antibodies involved in this analysis were previously reported (17, 54–56, 60–67). This analysis included the full sequence of the framework regions 1, 2, and 3 and CDR1 and −2. (B) Comparison of percent amino acid mutation between bNAbs (using the eight bNAbs presented in panel A) and anti-NHR antibodies; bNAbs have a mean somatic hypermutation of 25% compared to the 9.5% for anti-NHR antibodies. Each dot represents a unique antibody’s percent amino acid mutation, with error bars denoting standard errors of the means. The percentages of amino acid mutations from germline are reported on the *y* axis, as the published sequences cited above mostly reported only amino acid sequences.

Of relevance for vaccine applications, several monoclonal antibodies (mAbs) that target the NHR and inhibit HIV-1 infection have been characterized (53–57). The first of these mAbs was D5 (53), which was isolated from a human B cell-derived phage display library using two synthetic mimetics of the gp41 PHI: 5-helix (36) and IZN36 (58). X-ray crystallographic studies reveal that D5 binds a conserved hydrophobic pocket of the NHR (59). In contrast to most HIV-1 bNAbs (9, 60–66), D5 and other neutralizing mAbs that target the NHR (53–56, 67) contain far fewer amino acid changes resulting from SHM (Fig. 1). Thus, eliciting anti-PHI antibodies via vaccination is not expected to require extensive germline-targeting strategies. Indeed, various NHR-based vaccine candidates have been shown to elicit HIV-1-neutralizing antisera in animals (56, 68–70). These antisera, however, demonstrate only weak neutralization potency. Similarly, while exhibiting broad neutralizing activity, the potencies of mAbs targeting the NHR, including D5, are weak and mostly limited to tier-1 HIV-1 isolates (53–56). Taken together, these findings have led to understandable skepticism about the PHI as a vaccine target.

Here, we engineered more-potent versions of D5 by combining multiple mutations in the complementarity-determining region (CDR) loops of the antibody that were individually shown to increase the neutralization activity of D5 by Montgomery et al. (67). The most enhanced recombined variant, D5_AR, shows neutralization efficacy in several diverse tier-2 HIV-1 viruses. Thus, D5_AR presents proof of concept that an anti-PHI mAb, with low levels of SHM, can neutralize tier-2 HIV-1 viruses. In addition, as recently reported for D5 (71) and earlier for MPER mAbs (72, 73), the neutralization potency of D5_AR was enhanced ∼1,000-fold in target cells expressing the high-affinity immunoglobulin receptor FcγRI compared to those without. These results bolster attempts to target the PHI as an alternative and orthogonal approach toward an HIV-1 vaccine that is fundamentally different from the prevalent germline-targeting strategies to elicit bNAbs.

## RESULTS

### D5_AR, a recombined CDR mutant of D5, has enhanced neutralization potency against HIV-1 *in vitro*.

X-ray crystallography revealed that complementarity-determining region (CDR) loops in the antibody variable regions of both the heavy and light chains (VH and VL, respectively) contribute to the binding of D5 to the NHR (59). Informed by this insight, Montgomery et al. (67) sought to increase the neutralization potency of D5 by randomizing residues in five of the six CDRs (VH CDR1, −2, and −3 and VL CDR1 and −3). Four D5 IgG variants, each with only one CDR mutated, had slightly increased neutralization potency (67).

**FIG 2:**
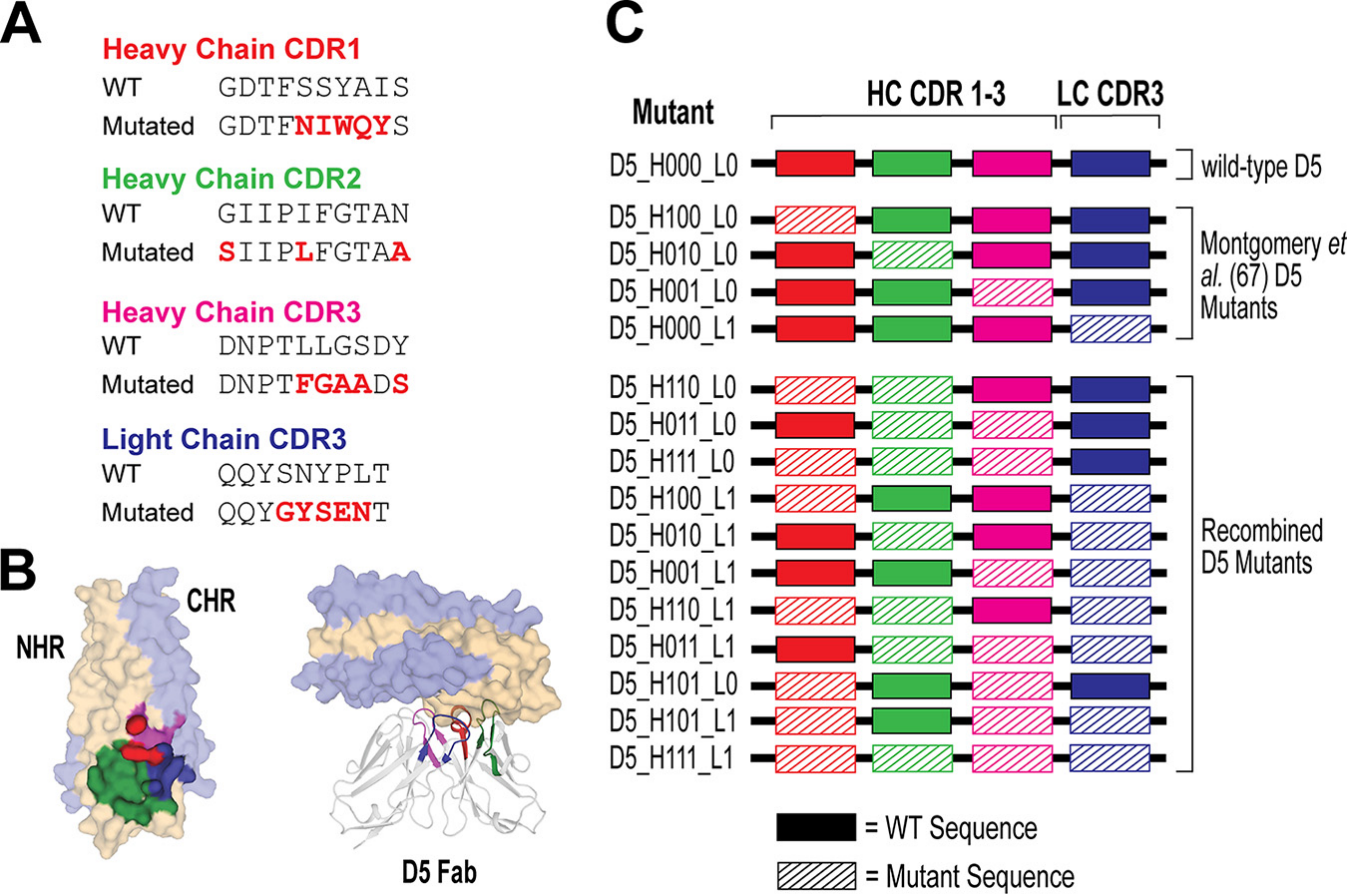
Recombination of known beneficial CDR sequences to engineer novel D5 variants. (A) Mutated CDR sequences of four highlighted D5 variants reported by Montgomery et al. (67) aligned with the wild-type (WT) sequence. These mutated CDR sequences were used to engineer the 11 recombined variants. (B) Left, paratope map of the binding sites for D5 Fab with its antigen, 5-helix. NHR, yellow; CHR, light blue. Areas of contact with the D5 CDR loops are represented as VH CDR1 (red), VH CDR2 (green), VH CDR3 (magenta), and VL CDR3 (blue). Right, X-ray crystal structure of D5 in complex with 5-helix (PDB 2CMR) (59). (C) Schematic of D5 variants engineered and tested. The identity of each of the four CDR loops is represented by 0 (WT) or 1 (mutant).

To assess the impact on neutralization after the introduction of multiple mutated CDRs, we recombinantly expressed and purified wild-type D5 IgG and 15 D5 IgG variants (Fig. 2). The variants were designated D5_HXXX_LX (Fig. 2C), with each X replaced by either 0 (representing the wild-type sequence) or 1 (representing the mutated CDR sequence) in the heavy (CDR1, −2, and −3) or light (CDR3) chain (Fig. 2A), reported by Montgomery et al. (67). These four CDRs form critical points of contact at the D5 epitope of the NHR (Fig. 2B). Using single-round infectivity assays in TZM-bl cells, each D5 IgG variant was screened for neutralization potency against lentivirus pseudotyped with HIV-1 HXB2 Env (74–78). Neutralization potency was reported as the 50% inhibitory dose (ID_50_). We confirmed that the four single-CDR mutants previously described by Montgomery et al. (67) (D5_H100_L0, D5_H010_L0, D5_H001_L0, and D5_H000_L1) displayed enhanced neutralization versus the parent D5.

Next, we screened 11 additional D5 variants with lentivirus pseudotyped with HIV-1 HXB2 Env for neutralization potency using a single-round infectivity assay. Several recombinant D5 variants had little effect or even diminished the neutralization potency compared to that of D5 (Table 1). Nevertheless, we identified six D5 variants that modestly enhanced (>2.0-fold) the neutralization potency of D5 (Table 1). Among these, D5_H011_L0, in which both CDR2 and CDR3 of the heavy chain are mutated, demonstrated the greatest enhancement (4-fold) in ID_50_ (Table 1). We renamed this enhanced D5 variant D5_AR.

**TABLE 1:** Neutralization profiles of D5 variants

Profile	D5 IgG variant	ID_50_ (μg/ml)^*a*^	Fold change^*b*^	*n*
Wild-Type	D5	48 ± 4.2	1.0	18
Modestly enhanced (>2.0-fold)	D5_H011_L0 (D5_AR)	12 ± 2.2	4.0 ± 0.78	6
	D5_H110_L0	20 ± 1.9	2.7 ± 0.57	4
	D5_H101_L0	20 ± 8.3	2.7 ± 0.34	3
	D5_H100_L0	31 ± 9.5	2.5 ± 0.84	2
	D5_H010_L0	35 ± 4.8	2.5 ± 0.12	2
	D5_H000_L1	33 ± 2.5	2.3 ± 0.78	2
Little to no effect (1.0- to 2.0-fold)	D5_H010_L1	42 ± 2.3	2.0 ± 0.79	3
	D5_H111_L0	43 ± 9.2	1.9 ± 0.41	4
	D5_H001_L0	54 ± 12	1.8 ± 0.36	2
	D5_H111_L1	62 ± 4.5	1.7 ± 0.68	3
	D5_H110_L1	28 ± 0.07	1.4 ± 0.03	2
	D5_H011_L1	80 ± 4.3	1.0 ± 0.06	2
Diminished neutralization (<1.0-fold)	D5_H100_L1	100 ± 9.8	0.47 ± 0.07	2
	D5_H101_L1	>300	NA	2
	D5_H001_L1	>300	NA	3

a The ID_50_ (half maximal inhibitory dose) of each D5 variant is represented by the geometric mean and standard error of the mean from replicate experiments.

b For each infection assay, the fold enhancement versus D5 was calculated (ID_50, D5_/ID_50, D5 variant_); reported fold enhancement is the geometric mean and standard error from replicate experiments. Fold enhancement of >1 corresponds to enhanced neutralization potency (reduced ID_50_). NA, not applicable.

**FIG 3:**
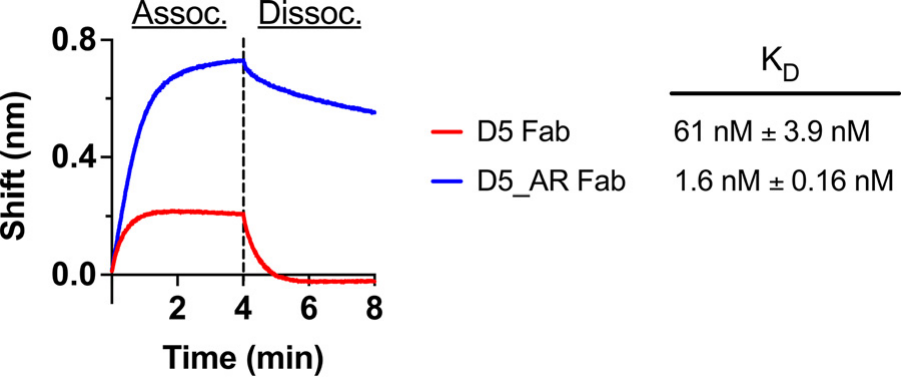
D5_AR Fab has a greater binding affinity to CCIZN17, an NHR-mimetic, than D5 Fab. The binding affinity (reported numerically as the *K_D_*) of D5 (red) and D5_AR (blue) Fab for CCIZN17, a variation of a previously described NHR mimetic (58, 79), is presented as association and dissociation curves using biolayer interferometry. The *K_D_*s are reported as the means from fitted values across multiple concentrations from at least two independent experiments and are reported with the standard errors of the means.

To further characterize D5_AR, we assessed the difference in binding for D5_AR Fab compared to D5 Fab against CCIZN17, a variation of a previously described mimetic of the NHR trimer (58, 79), using biolayer interferometry (see Materials and Methods). The Fab for D5_AR has ∼38-fold higher affinity for CCIZN17 than for D5 (1.6 nM for D5_AR compared to 61 nM for D5), providing a potential explanation for the enhanced neutralization activity of D5_AR (Fig. 3).

### Size exclusion chromatography purification impacts D5’s neutralization potency.

**FIG 4:**
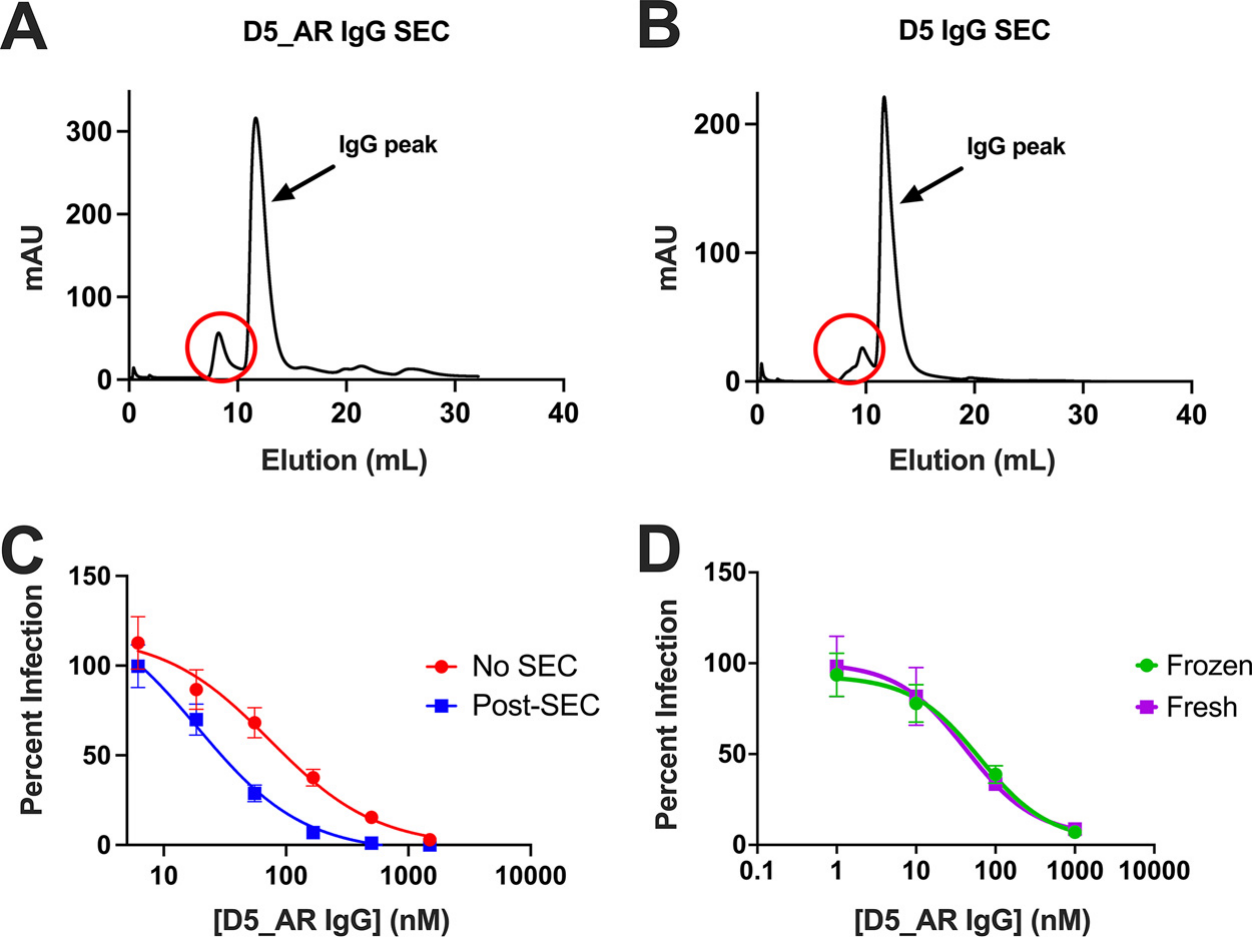
Purification via size exclusion chromatography (SEC) impacts the neutralization potency of D5_AR and reveals possible aggregation. (A and B) UV traces from SEC, performed after affinity chromatography, reveal possible protein aggregates (circled in red) for both D5 and D5_AR IgG. (A) The potential aggregate peak for D5_AR accounts for 11% of the total protein, while the major IgG fraction accounts for 83%. (B) Similarly, the aggregate fraction for D5 accounts for 8.2% of the total protein compared to the 90% of the major D5 IgG fraction. (C) After SEC purification, D5_AR IgG preparations demonstrated enhanced neutralization activity compared to that of a prep that was only purified via affinity chromatography. Each data point represents the mean percent infection with standard error of the mean (*n *= 3). (D) SEC-purified D5_AR IgG sample preps stored in 10% glycerol-1× PBS solution at −20°C retained similar neutralization activity after being thawed. Each data point represents the mean percent infection with standard error of the mean (*n *= 3).

Our initial neutralization screen (Table 1) utilized antibody purified via protein A affinity chromatography (see Materials and Methods). To obtain a more in-depth neutralization profile for D5_AR compared to that of D5, which included testing the constructs’ neutralization potency against various viruses and in different antibody formats, we added a size exclusion chromatography (SEC) step following protein A affinity chromatography. Notably, we found that the manner of purification of antibodies impacted the observed neutralization activity of the antibody (Fig. 4). A side-by-side comparison of a D5_AR antibody prep that was purified via only protein A chromatography with D5_AR that had the subsequent SEC purification step revealed that there was indeed a difference in neutralization for D5_AR (Fig. 4C); however, neutralization was still enhanced compared to that of D5. Reviewing the UV traces from SEC performed after affinity chromatography, we observed and removed aggregates for both D5 and D5_AR IgG (Fig. 4A and B). We note that the aggregate fraction in the SEC traces (Fig. 4A and B) accounted for 8.2% and 11% of the total protein for D5 and D5_AR, respectively; this is in comparison to the 90% and 83% in the major IgG fraction for D5 and D5_AR, respectively. We hypothesize that the presence of aggregates reduced the neutralization potencies of D5 and D5_AR, possibly because aggregated D5 has less neutralizing activity. Once aggregates were removed, D5 and D5_AR IgG preparations did not form aggregates readily. SEC-purified D5_AR samples frozen in 10% glycerol-1× phosphate-buffered saline (PBS) solution at −20°C retain similar neutralization activity after thawing (Fig. 4D).

Experiments in Table 1 were the only experiments reported here that used non-SEC-purified antibody preparations, which accounts for the difference in ID_50_ values for HXB2 reported in Table 1 (and see Fig. 6) (SEC-purified preparations had a 1.5-fold decrease in ID_50_ for D5 and a 2.3-fold decrease in ID_50_ for D5_AR).

### D5 and D5_AR neutralize with similar potency as an scFv, Fab, and IgG.

**FIG 5:**
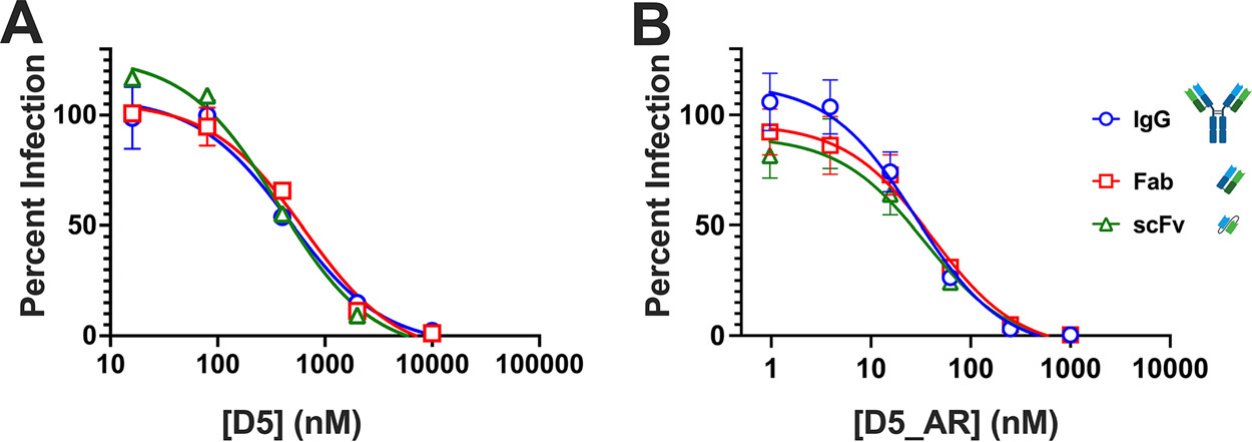
D5 and D5_AR neutralize HIV-1 *in vitro* in a size-independent manner. scFv, Fab, and IgG constructs of D5 (A) and D5_AR (B) are similarly effective *in vitro* at neutralizing lentivirus pseudotyped with HIV-1 HXB2. Data points and error bars are the mean percent infections and standard errors of the means, respectively (*n *= 2). Antibody construct images used in this figure were generated with BioRender.

In the first description of D5, the IgG (∼150 kDa) and single-chain variable-fragment (scFv) (∼25 kDa) constructs neutralized similarly to one another (53). However, more recent studies reported that D5 scFv is more potent than D5 Fab (∼50 kDa) and that both were more potent than D5 IgG (59, 67, 80); in addition, there are reports that increasing the size of NHR inhibitors reduces neutralization potency (55, 67, 80–82). These size-dependent findings would imply steric hindrance in accessing the PHI. Given our finding that SEC purification impacted the neutralization potency of D5 and D5_AR IgG (Fig. 4C), we decided to reinvestigate the question of size-dependent neutralization for D5 using antibody preparations free of aggregates. Notably, in agreement with the initial report (53) and in contrast to other reports (59, 67, 80), we found that D5 as an IgG, Fab, and scFv (all SEC purified) did not exhibit a size-dependent pattern of neutralization (Fig. 5A). This difference may be due to our final size exclusion chromatography step, which, to our knowledge, was not performed in other studies in which size dependence was observed. Additionally, we detected comparable neutralization potency for D5_AR as an scFv, Fab, and IgG (Fig. 5B). These results demonstrate that neither D5 nor D5_AR is impacted by steric hindrance and suggest the presence of protein aggregates could explain previous reports of size-dependent neutralization for D5 scFv, Fab, and IgG.

### D5_AR exhibits cross-clade tier-2 neutralization of HIV-1 viruses.

**FIG 6:**
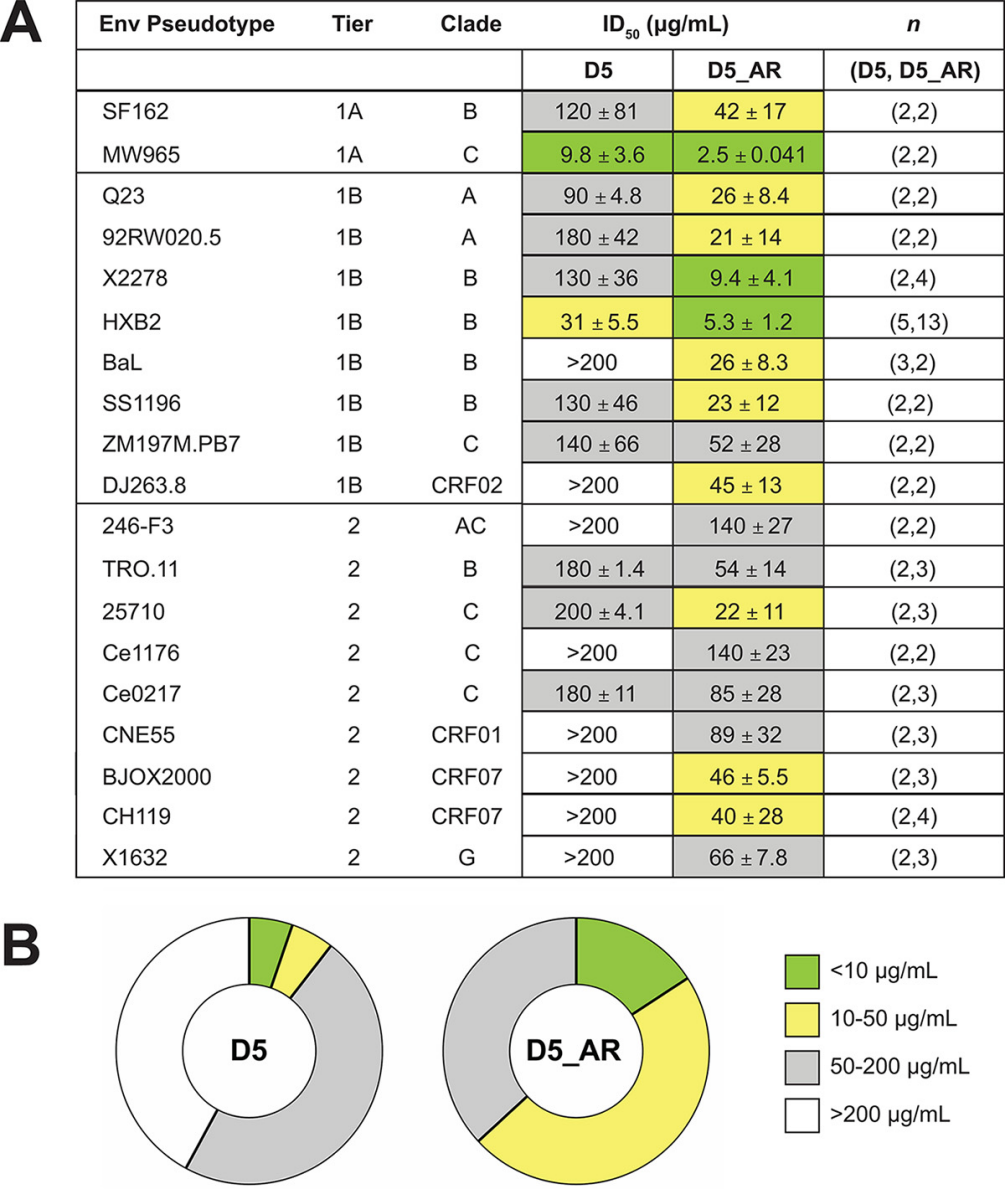
D5_AR IgG has higher neutralization potency than D5 across tiers and clades of HIV-1. (A) D5_AR IgG has an enhanced neutralization profile versus D5 IgG, as indicated by ID_50_ values (geometric mean ± standard error of the mean) against a panel of tier-1 and tier-2 HIV-1 viruses from multiple clades. (B) D5_AR IgG neutralizes a greater percentage of HIV-1 viruses than D5 IgG within the <10 μg/ml and 10 to 50 μg/ml ID_50_ ranges.

We next investigated the potency of D5_AR IgG in neutralizing a diverse panel of 19 tier-1 and tier-2 pseudotyped viruses across eight viral clades (A, AC, B, C, G, CRF01, CRF02, and CRF07) (77, 83–98). Ten of these strains originated from a 12-virus panel designed to capture the sequence diversity of the HIV-1 epidemic globally (77). Tier-1A and tier-1B contain viruses that are most sensitive to neutralization by antibodies, whereas tier-2 viruses have modest sensitivity to neutralizing antibodies (90). D5_AR IgG neutralized virus more effectively than D5 IgG across all tiers and clades (Fig. 6A). Notably, D5_AR neutralized more viruses with an ID_50_ of <50 μg/ml (63%) than D5 (11%) (Fig. 6B).

### D5_AR neutralization potency is enhanced 1,000-fold in FcγRI-expressing cells.

**FIG 7:**
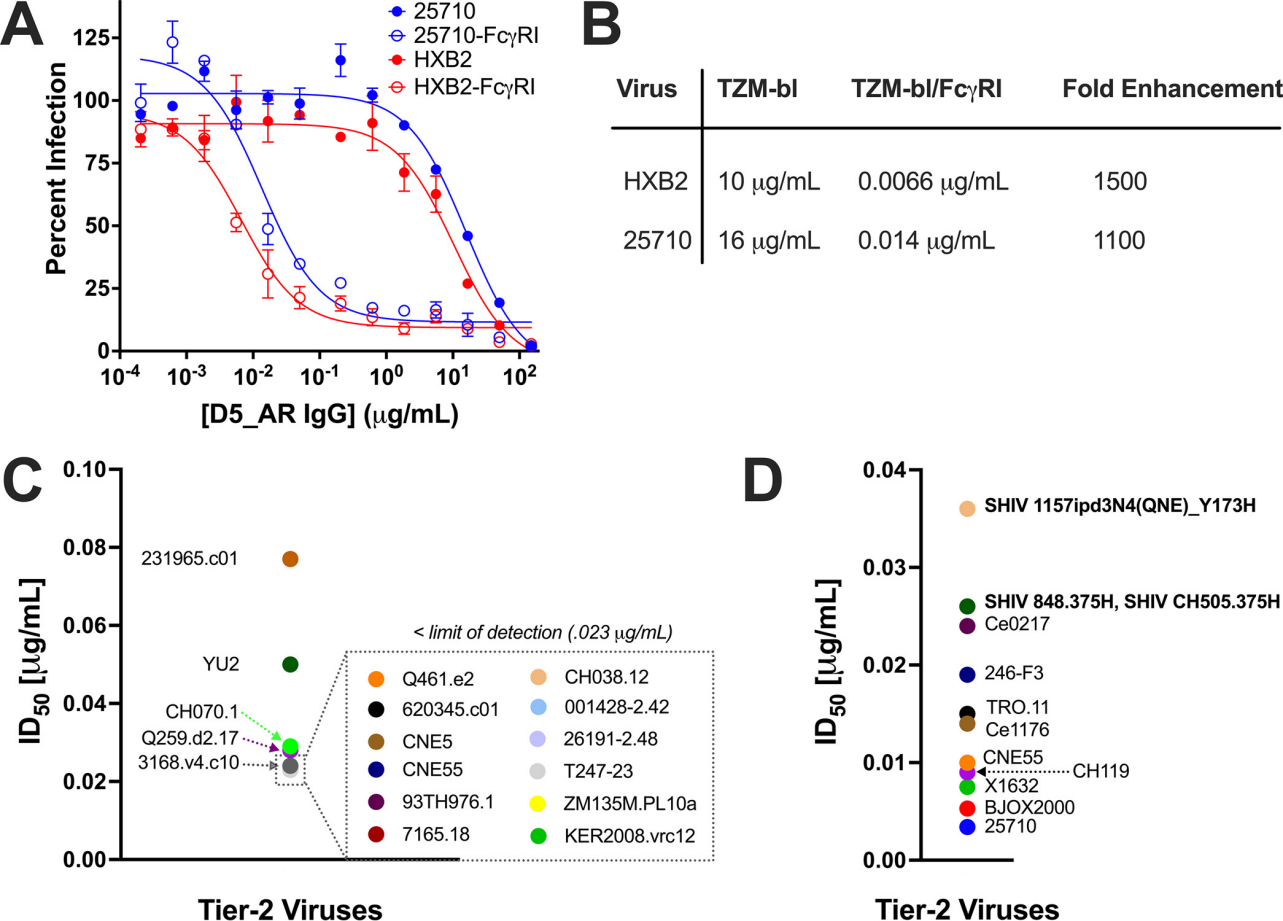
The neutralization potencies of D5_AR IgG against tier-2 HIV-1 viruses are substantially higher in TZM-bl cells expressing FcγRI. (A) Neutralization curves demonstrating the enhanced potency of D5_AR IgG against lentivirus pseudotyped with HIV-1 HXB2 (tier-1B) and 25710 (tier-2) in TZM-bl cells expressing FcγRI versus TZM-bl cells without FcγRI. Data points and error bars are means and standard errors of the means (*n *= 2), respectively. The neutralization curves presented in TZM-bl/FcγRI cells plateaued at around 90% neutralization compared to the 100% neutralization reported in TZM-bl cells; this is possibly explained by varying levels of FcγRI expression on the TZM-bl/FcγRI cells. (B) ID_50_ values and fold enhancement of neutralization potency of D5_AR IgG against lentivirus pseudotyped with HIV-1 HXB2 (tier-1B) and 25710 (tier-2) in TZM-bl cells versus that in TZM-bl/FcγRI cells. D5_AR is potentiated approximately 1,000-fold in TZM-bl/FcγRI cells. (C) ID_50_ values for D5_AR against a panel of tier-2 HIV-1 viruses in TZM-bl/FcγRI cells. Many of the viruses had ID_50_ values below the limit of detection (0.023 μg/ml). (D) ID_50_ values for D5_AR against another panel of tier-2 and SHIV challenge viruses in TZM-bl/FcγRI cells but with a lower limit of detection.

**FIG 8:**
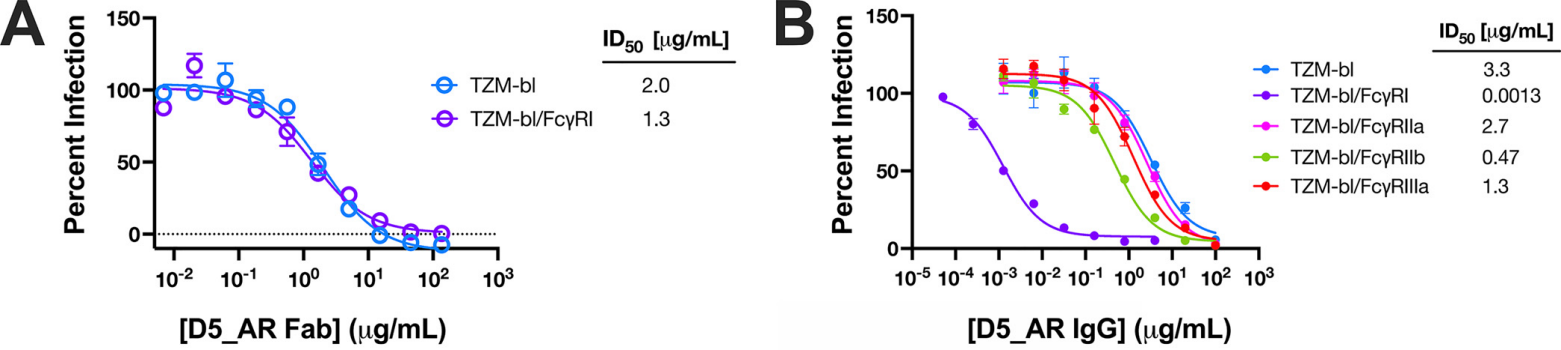
D5_AR is not potentiated as a Fab and shows minimal or no potentiation in the presence of other Fc receptors. (A) Neutralization curves for D5_AR Fab against lentivirus pseudotyped with HIV-1 HXB2 show no neutralization enhancement between TZM-bl cells and TZM-bl/FcγRI cells. Data points and error bars are the means and standard errors of the means (*n *= 2), respectively. (B) The degree of observed neutralization enhancement is FcγRI specific, as demonstrated by the neutralization potency of D5_AR IgG against lentivirus pseudotyped with HIV-1 HXB2 in TZM-bl cells expressing various Fc receptors. Data points and error bars are the means and standard errors of the means (*n *= 3), respectively.

**FIG 9:**
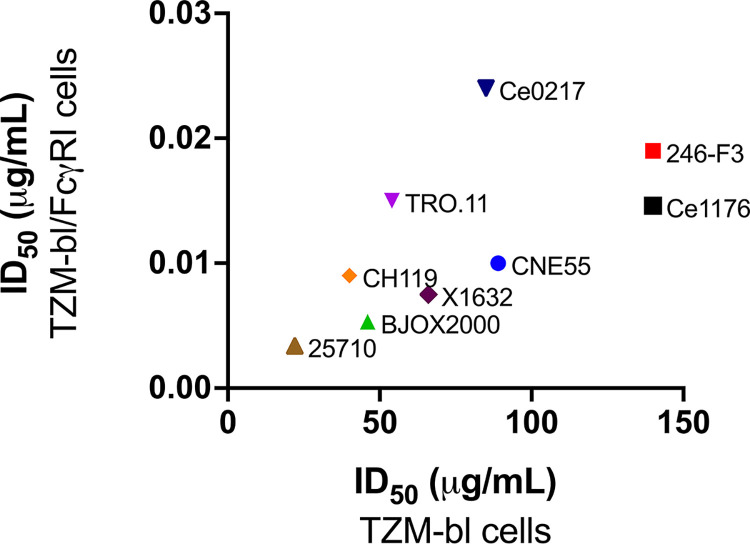
Comparison of HIV-1-neutralizing activity of D5_AR IgG in TZM-bl versus TZM-bl/FcγRI cells. The ID_50_ values of D5_AR IgG in TZM-bl and TZM-bl/FcγRI cells are approximately linearly related in a panel of tier-2 HIV-1 viruses from multiple clades. ID_50_ values represented in this figure were reported previously in Fig. 6 and 7 and selected because they are the only viral isolates for which we conducted neutralization analyses in both TZM-bl and TZM-bl/FcγRI cells.

Recently, we reported the neutralization potency of D5 IgG to be greatly increased in TZM-bl cells expressing the cell surface receptor FcγRI (TZM-bl/FcγRI cells) (71). FcγRI is the only known IgG receptor in humans capable of binding monomeric IgG with high affinity (99). To determine whether D5_AR IgG is similarly potentiated, we tested its neutralization of an additional panel of tier-2 HIV-1 viruses and SHIV challenge viruses in TZM-bl/FcγRI cells (Fig. 7). As recently reported for D5 (71), and as previously characterized for MPER mAbs (72, 73), the neutralization potency of D5_AR was enhanced at least 1,000-fold in TZM-bl/FcγRI cells compared to that in TZM-bl cells without FcγRI (Fig. 7A and B). In the presence of FcγRI, D5_AR had potent neutralization activity against a panel of tier-2 HIV-1 viruses, with ID_50_ values of <0.1 μg/ml (Fig. 7C and D). Consistent with an Fc-dependent mechanism, the Fab form of D5_AR did not exhibit potentiation (Fig. 8A). This observed potentiation was specific to FcγRI: enhanced neutralization of D5_AR IgG was minimal or not observed in cell lines expressing other Fc receptors (FcγRIIa, FcγRIIb, and FcγRIIIa) (Fig. 8B). It is noteworthy that the ID_50_ values of D5_AR IgG in TZM-bl cells were approximately linearly related to the ID_50_ values in TZM-bl/FcγRI cells (Fig. 9).

## DISCUSSION

The PHI of HIV-1 is a validated drug target in humans (50–52), and antibodies that bind the NHR of gp41 that is exposed in the PHI can inhibit HIV-1 infection *in vitro* (53–56, 67). The first of these NHR-binding antibodies, D5, has weak neutralization potency against tier-1 HIV-1 strains (53). We hypothesized that combining multiple CDR mutations from enhanced D5 variants previously described (67) would create a more-effective neutralizing mAb against HIV-1. To test this hypothesis, we evaluated a panel of 16 variants: wild-type D5, four previously described CDR variants (67), and 11 recombined CDR variants (Fig. 2). Here we engineered and characterized a more potent D5 derivative, D5_AR. Using 10 global HIV-1 reference strains, we determined that at modest concentrations, D5_AR neutralizes the majority of tier-1 and tier-2 HIV-1 strains tested from a variety of clades (Fig. 6). Like other anti-NHR antibodies (Fig. 1), D5_AR demonstrates a lower level of somatic hypermutation than bNAbs, with 7.6% of amino acids mutated from germline across both heavy- and light-chain V genes. Taken together with the high sequence conservation of the NHR (https://www.hiv.lanl.gov/content/sequence/HIV/mainpage.html), these proof-of-concept results suggest the PHI, and more specifically, the NHR, is a viable vaccine target.

Several previous reports had suggested that access to the D5 epitope was impacted by steric hindrance, as smaller antibody constructs were more potent than full-length IgG (55, 59, 67, 80). We reinvestigated this issue using antibody preparations that were free of observed protein aggregates (Fig. 5A). In contrast to earlier reports (59, 67, 80), here we confirm (53) that D5 and D5_AR are similarly potent when tested in neutralization assays in scFv, Fab, and IgG formats (Fig. 5). We hypothesize that the presence of higher-order protein aggregates (that can be removed by SEC) may explain the previous reports of size-dependent neutralization by D5. Given these findings for D5 and D5_AR, we conclude that steric hindrance is not an obstacle for at least some anti-PHI antibodies.

Previous work on antibodies targeting another epitope of gp41, the MPER, found that neutralization activity was potentiated as much as 5,000-fold in cells expressing FcγRI, an integral membrane protein that interacts with the Fc portion of γ immunoglobulins (72, 73, 100). Since the MPER is not fully exposed until after Env engages with cellular receptors (101, 102), these results suggest that by binding the Fc region of MPER mAbs, FcγRI provides a local concentration advantage at the cell surface that enhances neutralization (72, 73). Because the PHI, like the MPER, is fully exposed only during the viral membrane fusion process, we previously investigated the effect of FcγRI on D5 and found neutralization by this anti-PHI mAb is also enhanced ∼5,000-fold (71). Like D5, D5_AR IgG displayed ∼1,000-fold enhancement in neutralization potency in TZM-bl/FcγRI cells (Fig. 7A and B). In particular, we have previously demonstrated that D5 potency against HIV-1 HXB2 is enhanced ∼6,400-fold in the presence of FcγRI (71) compared to a range of ∼1,500-fold (Fig. 7B) to 2,500-fold (Fig. 8B) enhancement for D5_AR against HIV-1 HXB2. We conclude that the enhancement in FcγRI-expressing cells is independent of the effect of the mutations introduced in D5_AR. The magnitude of the potentiation observed by D5_AR is in line with what was previously reported for MPER antibodies and is thus much greater than antibodies to the CD4bs, V2 or V3 loop, or gp41 cluster, which show little to negligible enhancement in cells expressing FcγRI (72, 73). Notably, this enhancement makes D5_AR IgG an extremely potent neutralizing antibody of tier-2 HIV-1 viruses in the TZM-bl/FcγRI cell line, with ID_50_ values of <0.1 μg/ml (Fig. 7C and D).

TZM-bl/FcγRI cells enable sensitive detection of neutralization activity from anti-NHR antibodies and could be used, in conjunction with TZM-bl cells, to monitor progress toward eliciting neutralizing antisera that target the PHI. We hypothesize that neutralizing activity detected by TZM-bl/FcγRI cells could be used as an indicator of low-affinity antibody precursors in serum that have the potential to mature to high-affinity neutralizing activity independent of FcγRI. This hypothesis is supported by our findings that the neutralizing activity of D5_AR in FcγRI-expressing cells was approximately linearly related to the neutralizing activity of D5_AR in cells without FcγRI (Fig. 9).

Although not expressed on CD4^+^ T cells, FcγRI is expressed on macrophages and dendritic cells (100), which can be productively infected by HIV-1 (103–106), and can then mediate viral transmission to CD4^+^ T cells (107–110). Importantly, studies of intravaginal inoculation of simian immunodeficiency virus (SIV) of nonhuman primates demonstrated that intraepithelial and submucosal dendritic cells are infected in the earliest stages (18 to 48 h) of SIV infection (111–114). More recent work has shown that at 48 h postinoculation, 25% of infected cells are dendritic cells and macrophages, with the remainder comprising CD4^+^ T cells, primarily of the Th17 type (115, 116). Thus, it is plausible that inhibiting HIV-1 infection of these FcγRI-expressing cells at the mucosal surfaces by antibodies against the PHI could decrease the likelihood of sexual HIV-1 transmission (71). Indeed, in a vaginal challenge with SHIV in rhesus macaques, an MPER mAb (2F5) afforded dose-dependent protection when administered as an IgG but not when administered in its Fab form (117), suggesting an Fc-dependent mechanism of protection *in vivo*. Previous studies have also demonstrated that MPER mAbs are much more protective against SHIV challenge than other bNAbs when measured *in vitro* (118–121).

While D5_AR does not have the same breadth or potency as bNAbs, D5_AR demonstrates cross-clade tier-2 HIV-1-neutralizing activity and extremely potent activity when measured in cells expressing FcγRI (Fig. 7). These results motivate future efforts to investigate the ability of passively transferred anti-PHI antibodies such as D5_AR to protect against HIV-1 transmission in nonhuman primates. Importantly, D5_AR presents proof of concept that an anti-PHI MAb can neutralize tier-2 HIV-1 viruses. These results will encourage novel vaccine designs against the PHI as an alternative and orthogonal approach for HIV-1 vaccines that is fundamentally different from the prevalent approaches in the field of bNAb and germline-targeting strategies.

## MATERIALS AND METHODS

### Mammalian expression of D5 constructs.

The variable heavy (VH)-chain regions of the D5 variants were ordered as gene fragments from Twist Biosciences. Gene fragments were resuspended to 10 ng/μl in H_2_O and PCR amplified using the following two primers: (HC_forward) 5′-ACCGGTGTACATTCCCAGGTTCAAC-3′ and (HC_reverse) 5′-GCCCTTGGTCGACGCGCTTGATACG-3′. The mutated variable light (VL)-chain region, with only the third CDR mutated according to Montgomery et al. (67), was ordered as a gBlock gene fragment from Integrated DNA Technologies and PCR amplified using the following two primers: (LC_forward) 5′-ACCGGTGTACATTCAGATATTCAAATGAC-3′ and (LC_reverse) 5′-TGCAGCCACCGTACGTTTG-3′. Purified VH and VL fragments were cloned into linearized pCMVR with either the human IgG heavy- or kappa light-constant regions, respectively (9, 122). The primers for linearizing the pCMVR IgG heavy-chain plasmid were (HC_lin_forward) 5′-GCGTCGACCAAGGGCCCATCGGTCTTC-3′ and (HC_lin_reverse) 5′-GGAATGTACACCGGTTGCAGTTGCTACTAGAAAAAG-3′. The primers for linearizing the pCMVR IgG kappa light-chain plasmid were (LC_lin_forward) 5′-CGTACGGTGGCTGCACCATCTGTCTTCATCTTC-3′ and (LC_lin_reverse) 5′-TGAATGTACACCGGTTGCAGTTGCTACTAGAAAAAGGATGATA-3′. The D5 VH and VL segments were cloned into the linearized pCMVR backbones with 5× In-Fusion HD enzyme premix (Takara Bio). Plasmids were transformed into Stellar competent cells (Takara Bio), and transformed cells were grown at 37°C. Colonies were sequence confirmed and then maxiprepped (NucleoBond Xtra Maxi; Macherey-Nagel). Plasmids were sterile filtered using a 0.22-μm syringe filter and stored at −20°C.

D5 IgG variants used for neutralization assays were expressed in Expi293F cells (Thermo Fisher Scientific) using FectoPRO (Polyplus). VH and VL plasmids were cotransfected at a 1:2 ratio, respectively; cells were transfected at 3 × 10^6^ cells/ml. Cell cultures were incubated at 37°C and 8% CO_2_ with shaking at 120 rpm. Cells were harvested 3 days posttransfection by spinning at 300 × *g* for 5 min and then filtered through a 0.22-μm filter. IgG supernatants were diluted 1:1 with 1× phosphate-buffered saline (PBS) and batch bound to Pierce protein A agarose (Thermo Fisher Scientific) overnight at 4°C. The supernatant-resin slurry was added to a column, and the resin was washed with 1× PBS and eluted with 100 mM glycine (pH 2.8) into one-tenth volume of 1 M Tris (pH 8.0).

D5 and D5_AR Fab used for neutralization assays were also produced in Expi293F cells. D5 and D5_AR Fab VH regions were cloned into a pCMVR heavy-chain linearized backbone with a portion (CH2 and CH3 domains) of the constant region removed. Fab VH and VL plasmids were cotransfected and harvested with the protocol for IgG described above. Fab supernatants were diluted 1:1 with 50 mM sodium acetate (pH 5.0), batch bound to Pierce protein G agarose (Thermo Fisher Scientific) overnight at 4°C, washed with 50 mM sodium acetate (pH 5.0), and eluted with 100 mM glycine (pH 2.8) into one-tenth volume of 1 M Tris (pH 8.0).

The complete sequence of the variable heavy region (IGHV1-69 germline) for D5_AR IgG and Fab is QVQLVQSGAEVRKPGASVKVSCKASGDTFSSYAISWVRQAPGQGLEWMGSIIPLFGTAAYAQKFQGRVTITADESTSTAYMELSSLRSEDTAIYYCARDNPTFGAADSWGKGTLVTVSS. The complete sequence of the variable light-chain region (IGKV1-5 germline) for D5_AR IgG and Fab is DIQMTQSPSTLSASIGDRVTITCRASEGIYHWLAWYQQKPGKAPKLLIYKASSLASGAPSRFSGSGSGTDFTLTISSLQPDDFATYYCQQYSNYPLTFGGGTKLEIK.

D5 and D5_AR scFv constructs used for neutralization assays were expressed in Expi293F cells. The VH and VL regions were linked via a linker composed of 20 amino acids (ASTKGPSVKLEEGEFSEARV), tagged with a His_6_ tag, and cloned into a linearized pCMVR vector. The scFv plasmid was transfected and harvested with the same protocol as for IgG and Fab. scFv supernatants were diluted 1:1 with 10 mM imidazole in 1× PBS, batch bound to Ni-nitrilotriacetic acid (NTA) agarose (Thermo Fisher Scientific) overnight at 4°C, washed with 10 mM imidazole in 1× PBS, and eluted with 250 mM imidazole in 1× PBS.

The complete sequence of the D5_AR scFv insert is QVQLVQSGAEVRKPGASVKVSCKASGDTFSSYAISWVRQAPGQGLEWMGSIIPLFGTAAYAQKFQGRVTITADESTSTAYMELSSLRSEDTAIYYCARDNPTFGAADSWGKGTLVTVSSASTKGPSVKLEEGEFSEARVDIQMTQSPSTLSASIGDRVTITCRASEGIYHWLAWYQQKPGKAPKLLIYKASSLASGAPSRFSGSGSGTDFTLTISSLQPDDFATYYCQQYSNYPLTFGGGTKLEIKAAALEHHHHHH.

### Purification and storage of D5 constructs.

For the initial screening in neutralization assays (Table 1), there was no purification following elution from protein A affinity purification. Elutions were buffer exchanged and spin concentrated using 1× PBS and Amicon Ultra-15 10-kDa 15-ml spin concentrators (Millipore).

For all other neutralization assays, elutions were further purified after affinity purification on an AKTA using a GE Superdex 200 Increase 10/300 GL column (GE HealthCare) in 1× PBS. After size exclusion chromatography, samples were spin concentrated using Amicon Ultra-15 10-kDa 15-ml spin concentrators.

Fab and scFv constructs were eluted from affinity purification and then purified further via size exclusion chromatography using the Superdex 200 Increase 10/300 GL column (GE HealthCare) and 1× PBS. Samples were spin concentrated as described above.

For all samples, regardless of the purification procedure, concentrated elution samples were syringe filtered using a 0.22-μm filter and stored at 4°C prior to use.

### Synthesis of covalent biotinylated CCIZN17.

Biotinylated CCIZN17 (CCGGIKKEIEAIKKEQEAIKKKIEAIEKLLQLTVWGIKQLQARIL) was synthesized using standard Fmoc-based solid-phase peptide synthesis on a CSBio instrument. The resin was 250 μmol NovaSyn TGR R resin (Novabiochem), and coupling was performed for 15 min at 60°C with 4-fold molar excess of amino acids. Biotin was installed on the N terminus via coupling with biotin-polyethylene glycol (PEG)_4_-propionic acid (ChemPep). Dry peptide resin was cleaved with 94% trifluoroacetic acid, 2.5% water, 2.5% 1,2-ethanediol, and 1% triisopropylsilane at room temperature for 3.5 h followed by precipitation in cold diethyl ether. The crude peptide was purified by reversed-phase high-pressure liquid chromatography (HPLC) on a C_18_ semiprep column over an acetonitrile (ACN) gradient in the presence of 0.1% trifluoroacetic acid (TFA), and fractions were collected based on liquid chromatography-mass spectrometry (LC/MS) analysis. The pure monomeric protein was dissolved to 1 mg/ml in 100 mM Tris-HCl (pH 8.0) and oxidized by air at 37°C with gentle shaking for 48 h. The peptide mixture was then lyophilized, dissolved into 20% ACN-80% water, and repurified via HPLC. Peptide product whose mass corresponded to that of three biotinylated-CCIZN17 peptide chains linked by three-disulfide bridges was collected.

### Biolayer interferometry.

Biotinylated CCIZN17 peptide (200 nM) was loaded on streptavidin biosensors (Pall ForteBio) to a load threshold of 0.4 nm using an Octet RED96 system (Pall ForteBio). Sensors were immediately regenerated in 100 mM glycine (pH 1.5) and neutralized to remove aggregates and nonspecific interactions. Ligand-loaded sensors were dipped into known concentrations of Fab for an association step (4 min) and returned to the baseline well for a dissociation step (4 min). All reactions were run in PBS (pH 7.4) with 0.1% bovine serum albumin (BSA) and 0.05% Tween 20. All samples in all experiments were baseline subtracted to a well that loaded the tip with biotinylated peptide but did not go into the sample as a control for any buffer trends within the samples. Association/dissociation binding curves were fit in GraphPad Prism 8 using the “Association then Dissociation” analysis to calculate the equilibrium dissociation constant (*K_D_*), *k*_on_, and *k*_off_. Averages from fitted values across multiple concentrations from at least two independent experiments are reported.

### Transfection to produce HIV-1 pseudotyped lentiviruses.

HEK293T cells were transiently cotransfected with a backbone plasmid as well as an HIV-1 Env plasmid for HIV-1 pseudotyped lentivirus production using the calcium phosphate transfection protocol previously described (123–125). HEK293T cells were passaged in T75 flasks and incubated at 37°C at 5% CO_2_. The growth medium used for passaging and transfections was Corning Dulbecco’s modified eagle medium (DMEM; with 4.5 g/liter glucose, l-glutamine, and sodium pyruvate) with 10% fetal bovine serum, 1% penicillin-streptomycin (Corning), and 1% l-glutamine (Corning).

The backbone plasmid psg3ΔEnv was obtained through the NIH AIDS Reagent Program, Division of AIDS, NIAID, NIH from John C. Kappes and Xiaoyun Wu: HIV-1 SG3 ΔEnv noninfectious molecular clone (catalog number 11051) (126, 127). The psg3ΔEnv plasmid was propagated in MAX Efficiency Stbl2 cells grown at 30°C with shaking, and Env plasmids were propagated in Stellar competent cells grown at 37°C with shaking. DNA was isolated using a maxiprep kit (NucleoBond Xtra Maxi; Macherey-Nagel) and sequence confirmed.

In brief, 6 × 10^6^ HEK293T cells were plated in 10-cm petri dishes in a total volume of 10 ml of DMEM and incubated overnight at 37°C and 5% CO_2_ without shaking. Once the cells reached 50% to 80% confluence, they were transfected as follows. In a Falcon tube, 20 μg of psg3ΔEnv was mixed with 10 μg of Env plasmid and water for a final volume of 500 μl. Five hundred microliters of 2× HEPES-buffered saline (pH 7) (Alfa Aesar) was added dropwise to the mixture, and 100 μl 2.5 M CaCl_2_ was added subsequently. The mixture was incubated at room temperature for 20 min and then added dropwise onto the cells. Next, 12 to 18 h after transfection, the medium was aspirated from the dish and replaced with 10 ml of fresh DMEM with additives. Virus-containing medium was harvested 48 h after the medium swap and centrifuged at 300 × *g* for 5 min; the supernatant was sterile filtered with a 0.45-μm polyvinylidene difluoride filter and stored in 1-ml aliquots at −80°C.

### Neutralization assay.

The neutralization assay was adapted from the TZM-bl assay protocol using HIV-1 Env-pseudotyped viruses as described previously (75, 78). Briefly, TZM-bl cells, derived from the JC53-bl parental cell line, were used as reporter cells in this assay and were obtained through the NIH AIDS Reagent Program (catalog number 8129) from John C. Kappes and Xiaoyun Wu (126, 128–131). TZM-bl cells are adherent HeLa cells that stably express CD4 and CCR5 and constitutively express CXCR4; they have integrated β-galactosidase and firefly luciferase reporter genes under the control of the HIV-1 long terminal repeat (LTR) promoter. TZM-bl cells transduced to stably express FcγRI (72, 73) were also used in these neutralization assays. TZM-bl cells were passaged in T25 flasks and incubated at 37°C at 5% CO_2_ without shaking. The growth medium used for passaging and neutralization assays was Corning DMEM with 10% fetal bovine serum, 1% penicillin-streptomycin (Corning), and 1% l-glutamine (Corning).

In brief, 5 × 10^3^ TZM-bl cells were plated in the internal 60 wells of white-walled, clear-bottom 96-well plates and incubated overnight at 37°C at 5% CO_2_ without shaking. All outside wells were filled with 200 μl PBS to minimize evaporation. On the next day, the medium was aspirated without disturbing the cells and replaced with a final mixture composed of one-half volume DMEM, one-fourth volume HIV-1 pseudotyped lentivirus, one-fourth volume D5 antibody at varying concentrations, and DEAE dextran (10 μg/ml). Forty-eight hours after infection, all medium was aspirated off the wells, cells were lysed, and either luciferase activity was determined using BriteLite Plus reagent (Perkin Elmer) or β-galactosidase activity was determined using Tropix Gal-Screen (Applied Biosystems) and buffer A (Applied Biosystems). β-Galactosidase readout was used for neutralizations show in Table 1 and Fig. 4, 5, and 6. Luciferase readout was used for neutralizations shown in Fig. 7 and 8.

Relative luminescence unit (RLU) values were quantified using a Synergy HTX multimode reader (BioTek), normalized against cells-only reference wells, and averaged for technical replicates on the plate. Percent infectivity and propagated error values (see “Statistics and data analysis”) were entered into GraphPad Prism 8. Neutralization titers are reported as the antibody concentration at which RLUs were reduced by 50% compared to RLUs in virus control wells after subtraction of background RLU in cell control wells. ID_50_ was calculated using the inhibitor concentration versus response (three parameters) dose-response curve fit in GraphPad Prism 8. This assay was conducted in compliance with good clinical laboratory procedures (GCLP) (132), including participation in a formal TZM-bl assay proficiency program for GCLP-compliant laboratories (74).

### Statistics and data analysis.

Percent infectivity for the neutralization assays was calculated as follows:

$$\left(\frac{\text{sample RLU}-\text{cells-only RLU}}{\text{virus-only RLU}-\text{cells-only RLU}}\right)\times 100.$$

Propagated error for the percent infectivity was calculated using the following formula:

$$\left(\%\text{ infection}\right)\times \sqrt{{\left(\frac{\text{STD of sample RLU}}{\text{AVG of sample RLU}}\right)}^{2}+{\left(\frac{\text{STD of virus-only RLU}}{\text{AVG of virus-only RLU}}\right)}^{2}},$$

where STD is the standard deviation and AVG is the average. The ID_50_ values in Table 1 and Fig. 6 represent the geometric means from the biological replicates for the tested antibodies with the standard errors of the means reported. Fold difference in ID_50_ was calculated for each experiment by dividing the D5 ID_50_ by the D5 variant ID_50._ Because fold difference was calculated for each experiment, the reported fold differences in Table 1 are the geometric means and the standard errors of the means from all replicates.
